# On the Humoral Pathology

**Published:** 1825-05

**Authors:** William Stoker


Art. II.—*On the Humoral Pathology.* By William Stoker, M.D. (Addressed to "The Editors of the Medico-Chirurgical Review.") Communicated by Dr. James Johnson.

It was very gratifying to me to find that my work on Dropsy was deemed worthy of so much notice as was taken of it in your Journal; and I have been very desirous of making some acknowledgments for the kind expressions bestowed on myself on that occasion, as well as for such a public recognition of some of the facts which I had adduced in support of my opinion, "that substantial advantages might be derived for medical science, if the origin of diseased action in the living system was not considered so exclusively confined to the solid parts," as has for a long time been the prevailing hypothesis. Should the discussion thus commenced induce others to deviate from the beaten track, and endeavour to explore just pathological principles by the aid of actual observation and repeated experiments, I trust that the importance of these facts will be more fully established, as fixed and certain points to which those might safely revert who would escape the errors into which others have been led, either from the absence of certain lines of demarcation, which they had been taught to expect would define the object proposed, or from the glare of fanciful hypotheses, to which the sanguine and inexperienced more generally incline.

The marks of haste which the collection and arrangement of these facts and practical observations present, evince, I fear too obviously, that I was chiefly solicitous for their promulgation, confidently hoping that the value of the materials would not be overlooked, because of the want of symmetry in the structure. I have been thus long delayed from expressing my gratification, by having been, in a considerable degree, a sufferer in a certain debilitating form of the epidemic distemper prevailing in this country; a striking symptom of which form was insuperable aversion to bodily exertion or mental employment of any kind; and, even still, nothing less than the claims which my present task has on my exertions could induce me to undertake it.

Previously, however, to my entering on the few observations which I propose making on this occasion, I beg leave to disclaim any fastidious reluctance to submit to the censorship of the reviewers of my work; as the leading object of my reply is to avail myself of their just criticism, by trying to lesson or remove the obscurities appearing still to attach to those objects of research, which I wished to lay open to public view.

With a similar view, as well as to increase the array of facts adduced in support of the opinions I have ventured to express, I intend, with your permission, to subjoin a succinct account of certain forms of the epidemic, which I have before traced from the beginning of 1823, as they have been characterised in 1824, as well as during the current year; from which, I think, it will be found that most of the peculiarities which marked the rise of this formidable epidemic, in the latter end of the year 1822 and beginning of 1823, have ever since continued.

For the purpose of introducing in regular order the observations now proposed, I shall here, *in limine*, advert to an error (obviously a typographical one,) which occurs at the head of the review of my work on Dropsy, where it is stated that the date of its publication was March 1824,* instead of March 1823, as prefixed to the work itself.

Such an accidental mistake might not be deemed deserving of notice, but that, in the intervening year between the two dates just referred to, much attention has been bestowed on the subject of my work by some eminent physicians in England and in France, the results of whose numerous observations and experiments on the coagulation of the blood, and production of the buffy coat on it, though without any knowledge (as it appears) of my views, will be found to afford them corroboration. Slow coagulation is not mentioned by those writers as necessary to the production of the buffy coat; and more direct evidence is furnished that, in certain states of disease, this preternatural appearance is exhibited to the extent of those diseases, in whatever manner the blood is drawn,—whether from a large or a small orifice, or at a high or a low temperature; however these latter circumstances might, to a certain degree, seem to modify the results.

The conclusion which Dr. Scudamore deduces respecting the buffy appearance on the blood, is as follows:—"I am led, therefore, to the idea that, under such circumstances, the fibrin, instead of being distributed to the fibrous textures, as usual in health, remains in excess in the blood. The textures in question would be injured by receiving their usual supply of fibrin, being now in a state of disease; and, as regards the variation in the quantity of fibrin in different portions of blood drawn at the same time, it does not appear to me a difficult supposition, that, in the very short space of time occupied in venesection, the

---

* See *Medico-Chirurgical Review*, first vol. New Series, year 1824, article x.

state of circulation should change, so that the capillary arteries at once make a different distribution of the fibrin; resuming, in a great measure, their ordinary economy, and conveying it to the fibrous textures, instead of returning in the unnatural excess to the venous circulation. The fact itself of the different proportions of fibrin in the different cups of blood filled at the same venesection, is clearly proved. I know not any more probable theory to offer in explanation."*

To the first clause of the foregoing extract I am desirous of directing your attention, as it seems to me to be fairly deduced from experiment and observation; the rest of it being chiefly hypothetical. That fibrin in some diseases, and albumen in others, each perhaps imperfectly formed, is in excess in the blood, and causes the sizy surface which it exhibits after being drawn, fully accords with the opinions I have expressed on this subject: but the manner in which that excess is produced, has been differently explained by Dr. Scudamore and myself; as he supposes that, in such states of disease, the fibrous textures would be injured by receiving their usual supply of fibrin, which therefore accumulates in the blood; whilst I am of opinion that the excess of that substance which appears in the buffy coat, and which caused so much disturbance in the sanguiferous system, whilst in the blood-vessels during life, arises,—first, from the morbid condition of the chyle, as carried by the lacteal system, unfitting it for the necessary changes it should undergo in the minor circulation, or from diseased function in the lungs, unfitting them for the due sanguification of those matters carried thither by one of the chief avenues of supply;† and in this way the different kinds of surface which appear on the blood in diabetes and in pneumonia, might be accounted for; the milky-white appearance and loose texture of the one presenting so many of the characteristics of chyle, and the other of fibrin imperfectly assimilated. Secondly, there is a third kind of buffy coat, much firmer in consistence, deeper in colour, and occupying in general a much larger share of the coagulum: this appears to me to be produced either by the morbid condition of the venous blood, as it returns from the greater circulation by the vena portæ (the other great avenue of supply), or from disordered functions of the liver itself, preventing the due separation of the bile, which should take place in that organ, so as to refit the circulating fluid to be again prepared, in its transit through the minor circulation, to supply the waste of

---

* See *Essay on the Blood, &c. &c.* by CHARLES SCUDAMORE, M.D. F.R.S.—London, 1824.

† Vide *Examen du Sang, et de son Action dans les divers Phénomênes de la Vie,* par J. L. PREVOST, M.D. et J. A. DUMAS, Eléve en Pharmacie, Membre de la Sociêtè de Physique et d'Histoire Naturelle de Geneve.

substance, and perhaps of vitality, which is constantly going on in the animal frame.

The experiments lately made at the Hôtel Dieu, under the eye of Professor Recamier, on the coagulation and buffy coat of the blood, though with objects different from those with which I published, yet I think offer some confirmation of my opinions; and M. Belhomme, the Professor's experimenter, states that, in strongly inflammatory diseases, the buffy coat will appear, in whatever way the blood is drawn; and the curious fact which M. Belhomme notices, that blood drawn from pregnant women exhales an odour precisely like that which rises from a newly-extracted placenta, further evinces that the condition of the organ occupied in sanguification may impart certain characteristics of that condition to the blood in its transit.*

Whatever coincidence may be discovered in the results of the observations just alluded to, made by persons unknown to, and remotely distant from each other, I have been desirous thus to hail them as the commencement of the attention of physiologists to the chemistry of the fluids in the course of diseases; fully persuaded that, as the investigation advances, the importance of these observations will more fully and clearly appear. That such facts will be long viewed with jealousy, both by professors and practitioners, must be expected, from the great length of time during which the *modus operandi* of medicines, and the causes of death, have been deduced from opinions founded on anatomical inquiry. Indeed, so ample has been the field presented by anatomy to pathological inquiry, so luxuriant its produce, and so abundant the fruits of the labours of those engaged in its cultivation in every civilised country, that they may, with great justice, exclaim—

"Quæ regio in terris, nostri non plena laboris?"

It is no wonder, therefore, that it should be difficult to direct attention to a different soil; more especially as the humoral pathology, with which the chemistry of the fluids is so intimately connected, has still so much obloquy attached to its very name, owing to certain mistakes and pernicious errors of its followers, many of which anatomy has detected, and which are held up as a warning, rather than an invitation, to those who would look at any indication in that way.

If called on to adopt the doctrines of the humoral pathology as they were once taught, founded on the wild hypotheses of its enthusiastic followers, or to continue to be guided with as little reserve by those of the anatomists now in vogue, no one, qualified to estimate the relative advantages or disadvantages of the

---

* *Revue Medicale,* Mars 1824.

rival systems, could long hesitate to decide in favour of the latter. It is not *utrum horum mavis accipe.* The inquiry should be, whether, with the utmost care, a comparison between disorganised or altered structure, either in disease or in death, with that which is presented in the most perfect anatomical representations of the various parts of the body, in health and life, will in all cases be found to account, either for the *ratio symptomatum*, or suggest the *modus operandi* of medicines or the causes of death; this it will not do. Then let it be ascertained how far the results of observations made on the chemistry of the fluids in disease, will explain satisfactorily many points on which anatomy has failed; the successful issue of which latter inquiry is all I join in contending for, fully agreeing in the following remark on the creeds of the solidists and humoralists:—" Il ne serait pas moins deraisonnable d'adopter des idées toutes contraires, et de nier les alterations du sang, ou de les croire indifferentes aux phénomènes de la santè et de la vie. Un juste milieu entre ces deux extrèmes est, sans doute, la veritable route à suivre."*

I beg leave to place the following extract from the "Institutiones Medicæ" of Boerhaave, written in the beginning of the last century, as it appears to me to contain a true definition of pathology, and therefore such as will be again generally recognised, both in theory and practice.

"Qui itaque haberet perfectè intellectas omnes conditiones requisitas ad actiones, ille perspiceret clare defectum conditiones ex cognito morbo, et rursum bene caperet ex cognito defectum naturam morbi inde necessariò sequentis. Quæ scientia Παθολογία appelatur, inque suas partes dividitur.

"Ut itaque actiones, sic morbi, destingui possunt; ut conditiones ad actiones, ita et harum defectus: hinc 1. Morbi partis solidæ simplicis, organicæve; 2. Humorum morbi, horum naturam, copiam accidentia, spectantes; 3. Morbi ex his binis compositi, qui humani, masculi, fœmini, ad quas classes summas omnes compendio duci queunt."†

Some explanation for having introduced those two cases given in the preface of my work, appears, from the remarks of the reviewer, to be necessary to show their particular bearing on the subject; and I am the more inclined to give it, on account of the publicity you have been pleased to give to those cases, and because it will apply not only to the cases afterwards to be found in the work itself, but also, I hope, to those by which I intend to illustrate the course of the epidemic in 1824; in many

---

* *Revue Medicale*, Mars 1824.

† *Institutiones Medicæ in usus Annuæ Exercitationis Domesticos, digestæ ab* HERMANNO BOERHAAVE, page 304, anno 1773, Edinburgi.

forms of which, attention to the chemistry of the fluids could alone, as I conceive, suggest either the *ratio symptomatum* and *medendi*, or improvement in the *methodus medendi;* but, as I have already transgressed the limits to which I had intended to confine this letter, I hasten to conclude, and must defer, till another opportunity of addressing you, the explanation just alluded to, which shall consist of some reflections on the circumstances preceding these cases, their subsequent histories, and final results, as well as the other subjects I proposed entering on: and I have only to request that you will give this letter a place in the forthcoming Number of your Review, and that I may be allowed to transmit my next, in continuation of this subject, for insertion in a succeeding publication.

*York-street, Dublin; Feb. 24th,* 1825.

---

Art. III.—*On the Treatment of Wounds received during Dissection.* By John Shaw, Lecturer in Anatomy, &c.

